# Independent association of HbA1c and nonalcoholic fatty liver disease in an elderly Chinese population

**DOI:** 10.1186/1471-230X-13-3

**Published:** 2013-01-07

**Authors:** Han Ma, Chengfu Xu, Lei Xu, Chaohui Yu, Min Miao, Youming Li

**Affiliations:** 1 Department of Gastroenterology, the First Affiliated Hospital, College of Medicine, Zhejiang University, 79 Qingchun Road, Hangzhou, 310003, China; 2 Department of Gastroenterology, Ningbo No. 1 Hospital, Ningbo, China; 3 Department of Internal Medicine, Zhenhai Lianhua Hospital, Ningbo, China

**Keywords:** Fatty liver, Glycosylated hemoglobin, Elderly, Obesity

## Abstract

**Background:**

To investigate the association between serum glycosylated hemoglobin (HbA1c) levels and nonalcoholic fatty liver disease (NAFLD) in an elderly Chinese population.

**Methods:**

A cross-sectional study was performed among the 949 retired elderly employees of Zhenhai Refining & Chemical Company Ltd., Ningbo, China.

**Results:**

A total of 257 (27.08%) subjects fulfilled the diagnostic criteria of NAFLD, and NAFLD patients had significantly higher serum HbA1c levels than controls (*P* <0.001). The prevalence of NAFLD was significantly higher in subjects with increased serum HbA1c level (HbA1c ≥6.5%) than in those with normal range of serum HbA1c level (51.71% *vs.* 25.20%; *P* <0.001), and the prevalence increased along with progressively higher serum HbA1c levels (*P* for trend <0.001). Stepwise logistic regression analysis showed that serum HbA1c level was significantly associated with the risk for NAFLD (odds ratio: 1.547, 95% confidence interval: 1.054 – 2.270; *P* =0.026).

**Conclusions:**

Our results suggest that serum HbA1c level is associated with NAFLD, and increased serum HbA1c level is an independent risk factor for NAFLD in elderly Chinese.

## Background

Nonalcoholic fatty liver disease (NAFLD) has become one of the most common chronic liver disease worldwide, and the condition affects one in four adults in the general population [1,2]. The spectrum of NAFLD is wide ranging from simple steatosis through nonalcoholic steatohepatitis (NASH) to cirrhosis [3]. Patients with simple steatosis appear to have benign progression, while patients with NASH may progress to cirrhosis and even hepatocellular carcinoma [4].

NAFLD is closely associated with the metabolic syndrome, a cluster of metabolic abnormalities including central obesity, hypertension, dyslipidaemia and type 2 diabetes [5,6]. The metabolic syndrome, partly through glucose intolerance, is linearly linked with hepatic steatosis, fibrosis and cirrhosis, suggesting close connection between glycemic level and NAFLD [7]. In addition, the prevalence of NAFLD increases as the increase of glycemic level [8]. Glycemic ranges can be assessed by many methods, including glycosylated hemoglobin (HbA1c). HbA1c, reflecting average glycemia over the preceding 8 –12 weeks, is used to estimate chronic glycemic levels with 6.5% as the cutoff point to diagnose diabetes [9]. However, when it comes to HbA1c, the association between NAFLD and glycemic levels has not been well documented so far.

NAFLD mainly occurs among middle-aged to older people, with a mean presentation age of 44–50 years [10,11]. However, Koehler *et al.* observed that the prevalence of NAFLD was as high as 35.1% in the elderly [12]. Recent studies also showed that age was an independent risk factor for NAFLD development [4,13]. A large sample cross-sectional analysis revealed an independent association between HbA1c level and NAFLD in predominantly middle-aged subjects [14]. Considering serum HbA1c level increases with age [15], middle-aged participants may probably not well represent the elderly population and not fully highlight the significance of HbA1c in NAFLD. For these reasons, clarifying the association between HbA1c level and NAFLD in elderly subjects may have significant clinical implications for the diagnosis and prevention of NAFLD by monitoring HbA1c level.

Here, we performed a cross-sectional study to investigate the association between HbA1c and NAFLD determined by ultrasonography in an elderly Chinese population.

## Methods

### Study design and subjects

This study was conducted among retired elderly employees (age ≥65 years) of Zhenhai Refining & Chemical Company Ltd. (Ningbo, China). All of the retired elderly employees who attended their annual health examination during the year of 2010 were initially enrolled. The following subjects were excluded: subjects with alcohol consumption greater than 140 g/week for males and 70 g/week for females, or subjects with a history of viral hepatitis, autoimmune hepatitis, or other forms of chronic liver disease. Moreover, subjects who were previously diagnosed as either diabetes or anemia were also excluded. The definition of anemia in our study was serum hemoglobin <120 g/L for males and < 110 g/L for females. A total of 949 eligible subjects were enrolled (603 males and 346 females, with mean age of 71.5 ± 4.5 years). Verbal informed consent was obtained from each subject and was recorded by the physician who explained the study procedures. Written informed consent was not required because of the observational nature of the study. This study protocol was approved by the Ethics Committee of the First Affiliated Hospital, College of Medicine, Zhejiang University, and was in compliance with the Helsinki Declaration.

### Clinical examination

The clinical examinations were performed as described previously [16,17]. In brief, all subjects were required to refrain from exercise for one day prior to the examination. Systolic and diastolic blood pressures were measured by standard clinical procedures. Standing height, body weight and waist circumference were recorded for all subjects. Waist circumference was measured with the measuring tape positioned midway between the lowest rib and the superior border of the iliac crest as the patient exhaled normally. Body mass index (BMI) was calculated as weight divided by height squared and was used as the criteria for diagnosis of overweight and obesity.

### Biochemical analyses

Fasting blood samples were obtained for the analysis of biochemical values and HbA1c without frozen. The biochemical values included liver enzymes, lipids, uric acid and glucose. All biochemical values were measured by an Olympus AU640 autoanalyzer (Olympus, Kobe, Japan) using standard methods. Serum HbA1c levels were assessed by affinity chromatography method.

### Diagnosis of NAFLD and the metabolic syndrome

The diagnosis of NAFLD was based on the criteria suggested by the Chinese Liver Disease Association [18]. Ultrasonic examination was carried out by a trained ultrasonographist who was unaware of the results of physical examination and biochemical analyses. Diffuse fatty liver can be defined by abdominal ultrasonography with the presence of at least two of three findings below: ‘bright liver’, liver echo greater than kidney, vascular blurring and the gradual attenuation of far field ultrasound echo [18]. The examination was performed by using a Toshiba Nemio 20 sonography machine with a 3.5-MHz probe (Toshiba, Tokyo, Japan). The diagnosis of the metabolic syndrome was based on the criteria suggested by the new International Diabetes Federation definition [19].

### Statistical analyses

Statistical analyses were performed using SPSS 13.0 for Windows (SPSS Inc., Chicago, IL). The Kolmogorov-Smirnov test was used to assess whether continuous data were normally distributed. Continuous variables are presented as mean and standard deviation (SD) or median and interquartile range (IQR) according to the distribution of data. Student’s *t*-test or the Mann–Whitney *U* test was used for comparisons of continuous data, while chi-squared test was used for comparisons of categorical variables. Adjusted regression analysis was used to investigate the relationship between HbA1c level and prevalence of NAFLD. Furthermore, stepwise logistic regression analysis (Backward: Wald; Entry: 0.05, Removal: 0.10) was used to evaluate the risk factors for NAFLD. *P* <0.05 (2-tailed test) was considered statistically significant.

## Results

### Characteristics of study subjects

Among the 949 enrolled subjects, 257 (161 males and 96 females) met the diagnostic criteria for NAFLD; the prevalence of NAFLD was 27.08% (26.70% and 27.75%, in males and females, respectively). A total of 212 subjects (22.34%) met the diagnostic criteria for the metabolic syndrome. The prevalence of the metabolic syndrome components, including central obesity, hypertriglyceridemia, low HDL cholesterol, elevated blood pressure and elevated FPG were 49.53%, 25.71%, 13.80%, 74.71% and 17.39%, respectively.

The characteristics of the subjects, classified by the presence or absence of NAFLD, were presented in Table 1. Subjects with NAFLD had higher BMI, waist circumference, systolic and diastolic blood pressure, γ-Glutamyltransferase, triglyceride, fasting plasma glucose, serum uric acid levels, and a lower HDL cholesterol level than subjects without NAFLD. As noticed in Table 1, significant higher serum HbA1c level was observed in the subjects with NAFLD than those without NAFLD (Table 1).

**Table 1:** Characteristics of study subjects according to presence of NAFLD

**Variables**	**NAFLD present (n = 257)**	**NAFLD absent (n = 692)**	***t*value**	***P*value**
Age (year)	71.22 (4.34)	71.55 (4.53)	1.003	0.316
Gender (male/female, n)	161/96	442/250	0.122^a^	0.762
Body mass index (kg/m^2^)	26.79 (2.86)	23.48 (2.67)	16.612	< 0.001
Waist circumference (cm)	91.2 (8.5)	83.6 (8.1)	12.773	< 0.001
Systolic blood pressure (mmHg)	141.7 (16.9)	136.6 (18.2)	3.878	< 0.001
Diastolic blood pressure (mmHg)	81.9 (9.9)	79.1 (10.3)	3.816	< 0.001
γ-Glutamyltransferase (U/L)	27.0 (20.0 – 41.0)	20.0 (15.0 – 28.0)	8.484 ^b^	< 0.001
Triglyceride (mmol/L)	1.60 (1.25 – 2.06)	1.13 (0.85 – 1.52)	10.337^b^	< 0.001
Total cholesterol (mmol/L)	5.12 (0.98)	5.14 (0.94)	0.343	0.732
HDL cholesterol (mmol/L)	1.32 (0.26)	1.49 (0.32)	7.532	< 0.001
LDL cholesterol (mmol/L)	2.93 (0.85)	3.01 (0.78)	1.392	0.164
Fasting plasma glucose (mmol/L)	5.19 (4.75 – 5.66)	4.92 (4.63 – 5.31)	5.225^b^	< 0.001
Serum uric acid (μmol/L)	363.9 (87.2)	327.0 (83.5)	5.976	< 0.001
Serum HbA1c (%)	5.80 (5.50 – 6.10)	5.60 (5.40 – 5.90)	5.733^b^	< 0.001

Data are expressed as mean (SD) or median (IQR). ^a^*χ*^2^ value; ^b^*Z* value; HDL, high-density lipoprotein; LDL, low-density lipoprotein.

### Association of HbA1c level with prevalence of NAFLD

Among the 949 enrolled subjects, 68 (7.17%) had increased serum HbA1c level (HbA1c ≥6.5%), while the remaining 881 (92.83%) had normal range of serum HbA1c level (HbA1c <6.5%). The prevalence of NAFLD was much higher in the subjects with increased serum HbA1c level than in those with normal range of serum HbA1c level (51.71% *vs.* 25.20%; *χ*^2^ test, *P* <0.001).

**Figure 1:**
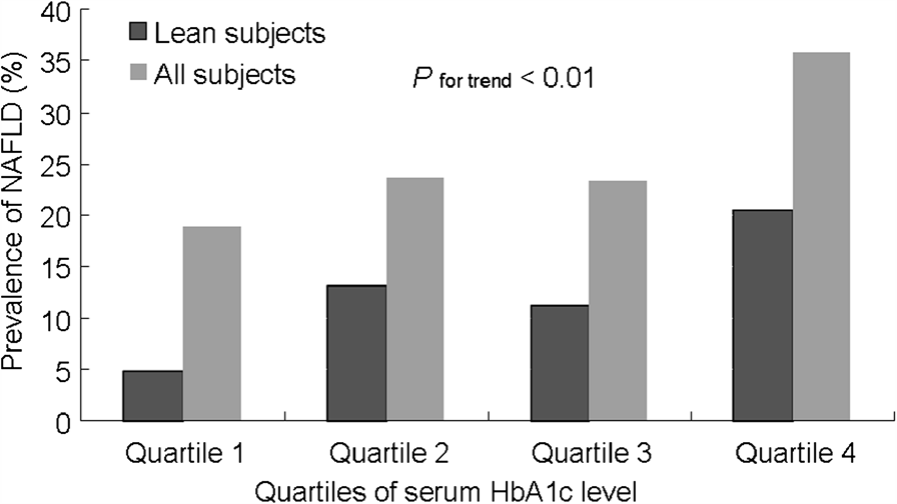
**Prevalence of NAFLD associated with normal range of serum HbA1c levels.** The prevalence of NAFLD was positively correlated with increase in normal range of serum HbA1c level.

To investigate whether normal range of serum HbA1c was also associated with prevalence of NAFLD, subjects with normal range of serum HbA1c were further divided into quartiles as follow: quartile 1: HbA1c ≤5.4%, quartile 2: 5.4% < HbA1c ≤5.6%, quartile 3: 5.6% < HbA1c <5.9%, and quartile 4: 5.9% ≤ HbA1c <6.5%. As showed in Figure 1, the prevalence of NAFLD was positively associated with serum HbA1c level. The prevalence of NAFLD was 18.84% for the subjects with serum HbA1c level in quartile 1, and the prevalence increased to 35.91% for the subjects with serum HbA1c level in quartile 4 (Figure 1; *P* value for trend <0.01). This indicated that change of normal range of serum HbA1c was also associated with prevalence of NAFLD.

### Independent association between HbA1c level and NAFLD

Obesity and the metabolic syndrome are two major factors that closely associated with NAFLD, and these two factors may act as cofactors for the link between serum HbA1c level and NAFLD. To explore whether the association between serum HbA1c level and NAFLD was independent of obesity and the metabolic syndrome, 470 subjects with obesity and/or the metabolic syndrome were excluded for further analysis. Among the remaining 479 lean subjects, 59 fulfilled the diagnostic criteria for NAFLD, 21 had increased serum HbA1c level. The prevalence of NAFLD was also significantly higher in the subjects with increased serum HbA1c level than in those with normal range of serum HbA1c level (28.57% *vs.* 11.57%; *χ*^2^ test, *P* =0.033).

To investigate whether normal range of serum HbA1c was also associated with prevalence of NAFLD in lean elderly subjects, 458 lean subjects with normal range of serum HbA1c were further divided in to quartiles as described above. As showed in Figure 1, the prevalence of NAFLD was also positively correlated with serum HbA1c level. These results not only suggested that the link between serum HbA1c level and NAFLD observed from the general elderly population also holds true for lean elderly subjects, but also indicated that the link might probably not influenced by other metabolic disorders.

### Serum HbA1c level and risk factor of NAFLD

Logistic regression analysis was used to evaluate the risk factors for NAFLD. Sixteen variables, including age, gender, weight, height, BMI, waist circumference, systolic and diastolic blood pressure, γ-Glutamyltransferase, total cholesterol, triglyceride, HDL and LDL cholesterol, fasting plasma glucose, serum uric acid and HbA1c level were entered into the analysis. Linear regression was used to evaluate the collinearity among the variables. LDL cholesterol and total cholesterol had a high variance proportions in this analysis indicating a high level of collinearity between these LDL cholesterol and total cholesterol. Thus 15 variables, with LDL cholesterol and total cholesterol as a whole, were entered into the initial equation of stepwise logistic regressions. Our result showed that age, gender, BMI, waist circumference, γ-Glutamyltransferase, triglyceride, HDL cholesterol, fasting plasma glucose, serum uric acid and HbA1c were significantly associated with the risk for NAFLD (Table 2). A notable finding was that serum HbA1c level was found to be significantly associated with risk factor for NAFLD (OR: 1.547, 95% CI: 1.054 – 2.270; *P* =0.026).

**Table 2:** Risk factors associated with the presence of NAFLD

**Variables**	**β**	**SE**	**Wald*χ***^**2**^	***P*value**	**OR (95% CI)**
Age (year)	−0.056	0.021	7.294	0.007	0.945 (0.908 – 0.985)
Male gender	−0.779	0.241	10.406	0.001	0.459 (0.286 – 0.737)
Body mass index (kg/m^2^)	0.352	0.048	53.123	<0.001	1.422 (1.293 – 1.563)
Waist circumference (cm)	0.035	0.017	4.436	0.035	1.036 (1.002 – 1.071)
γ-Glutamyltransferase (U/L)	0.011	0.004	8.066	0.005	1.011 (1.003 – 1.019)
Triglyceride (mmol/L)	0.523	0.121	18.525	<0.001	1.686 (1.329 – 2.139)
HDL cholesterol (mmol/L)	−1.589	0.379	17.609	<0.001	0.204 (0.097 – 0.429)
Fasting plasma glucose (mmol/L)	0.262	0.141	3.439	0.064	1.299 (0.985 – 1.713)
Serum uric acid (mmol/L)	0.004	0.001	9.514	0.002	1.004 (1.001 – 1.006)
Serum HbA1c (%)	0.436	0.196	4.969	0.026	1.547 (1.054 – 2.270)

β, partial regression coefficient; SE, standard error of partial regression coefficient; OR, odds ratio; CI, confidence interval; HDL, high-density lipoprotein.

## Discussion and conclusion

Our results showed that serum HbA1c was independently associated with NAFLD in an elderly Chinese population. First, serum HbA1c level was significantly increased in NAFLD patients, and the level was positively correlated with prevalence of NAFLD. Second, subgroup analysis indicated that the correlation between serum HbA1c level and NAFLD was independent of obesity and the metabolic syndrome. Third, the result of logistic analysis showed that increased serum HbA1c level was significantly associated with risk factor for NAFLD.

This relationship can be explained by the following two reasons. One of the possible explanations for the increased HbA1c in NAFLD patients is insulin resistance. Insulin resistance is a central component of NAFLD [20]. Hepatic steatosis played an important role in the pathogenesis of insulin resistance. Recent studies indicated that impaired hepatic lipid and lipoprotein settling and increased oxidative stress in liver cells may increase liver fat accumulation and result in insulin resistance [21,22], thus increase the hepatic glucose production and export to the peripheral circulation, as a result, raise the level of serum glucose [23]. The level of HbA1c is influenced by lifespan and “glucose permeability” of the erythrocytes [24]. As indicated by a recent study, transmembrane glucose gradient rather than the rate of glucose transport correlates with the HbA1c [25]. Based on this point, elevated HbA1c in NAFLD in this study can be stated as the increase of intracellular glucose in NAFLD patients.

The other possible mechanism by which HbA1c linked with NAFLD is through oxidative stress. Oxidative stress is a key pathophysiological process responsible for NAFLD [26]. In response to oxidative stress, erythrocytes may undergo morphology change and decreased membrane fluidity, becoming easy to be captured by liver macrophages [27]. An enhanced erythrocytes susceptibility to haemolysis was reported in an experimental model of NAFLD and in obese subjects [28]. Immoderate haemolysis will in return enhance oxidative stress [29]. As red blood cell fragility, which influences lifespan of the erythrocytes, was positively correlated with glycosylated hemoglobin [24,30], oxidative stress should be a likely explanation.

The link between HbA1c and NAFLD may provide a potential explanation for why NAFLD can be a risk for cardiovascular diseases. Independent association between NAFLD and cardiovascular disease was emphasized in several epidemiological studies [31,32]. However, the physiological mechanism for the link between NAFLD and cardiovascular disease remains unclear. Since HbA1c is indicated to be a marker of cardiovascular risk [33,34], the elevated HbA1c reported in elderly NAFLD patients in the present study may partially interpret why NAFLD increases the risk for cardiovascular disease. According to this view, strategies that aim at decreasing serum HbA1c level in order to reduce risk of cardiovascular disease in NAFLD patients may be taken into account.

Our study has several limitations. First, the diagnosis of NAFLD was based on ultrasonographic examination. Although ultrasonography is non-invasive, reasonably accurate and widely used in epidemiological studies of NAFLD, it is not sensitive enough to detect mild steatosis. In addition, HbA1c cutoff points for the identification of the severity of NAFLD can not be identified in this study. Moreover, the question about whether or not elevated HbA1c is a bystander, a cause or a consequence of NAFLD is unresolved in our study. Further researches are needed to reveal the detailed relationship and the possible mechanisms between serum HbA1c and NAFLD.

In conclusion, our results showed that serum HbA1c level was significantly correlated with NAFLD in elderly Chinese. Further clarify the precise relationship may have significant clinical implications for the diagnosis and prevention of NAFLD by monitoring HbA1c level.

## Competing interests

The authors declare that they have no competing financial interests.

## Authors’ contributions

HM, CFX, CHY, LX, MM and YML designed and conducted the study. HM and CFX performed the statistical analysis and drafted the manuscript. LX, CHY and MM collected the data. All authors read and approved the final manuscript.

## Pre-publication history

The pre-publication history for this paper can be accessed here:

http://www.biomedcentral.com/1471-230X/13/3/prepub
